# Epidemiological surveillance of childhood and adolescent malignancies as a tool for public health strategy: insights from the Polish National Cancer Registry 2023

**DOI:** 10.3389/fpubh.2026.1898195

**Published:** 2026-07-31

**Authors:** Michalina Irzyk, Patrycja Filipek, Urszula Wojciechowska, Anna Raciborska, Andrzej Kurylak, Joanna Aleksandra Didkowska

**Affiliations:** 1 Polish National Cancer Registry, Maria Sklodowska-Curie National Research Institute of Oncology, Warsaw, Poland; 2 Department of Oncology and Surgical Oncology for Children and Youth, Institute of Mother and Child, Warsaw, Poland; 3 Department of Pediatrics, Oncology, Hematology and Rheumatology, Provincial Children's Hospital in Bydgoszcz, Bydgoszcz, Poland

**Keywords:** adolescents, cancer registry, childhood cancer, epidemiology, incidence, Poland

## Abstract

**Background:**

Pediatric malignancies require specialized epidemiological surveillance due to their distinct developmental etiology and unique societal impact. In the context of contemporary European demographic trends, characterized by sub-replacement fertility, monitoring health outcomes in younger cohorts has become a strategic public health priority.

**Methods:**

This population-based retrospective analysis evaluates the incidence and epidemiological patterns of childhood and adolescent malignancies in Poland in 2023, utilizing Polish National Cancer Registry (PNCR) data to provide an evidence base for health policy optimization.

**Results:**

Among the 1,229 new cases recorded (782 in the 0–14 cohort), a bimodal age distribution peaked at 0–4 and 15–19 years. While the aggregate gender burden was balanced [Incidence Rate Ratio (IRR) = 1.01], significant biological disparities occurred in specific subtypes, notably a marked female predominance in thyroid carcinoma and an extreme male predilection in Burkitt lymphoma (IRR = 10.43 in the 0–14 group). Descriptive long-term trend analysis suggests an upward trajectory in incidence, with an observed localized peak in 2021–2022.

**Discussion:**

Given current demographic constraints, with Poland's fertility rate (1.2) significantly below the EU average, these malignancies represent a disproportionate loss of future human capital. Our findings underscore that pediatric oncological care is a high-efficiency investment with a substantial social return. We advocate for the establishment of a dedicated pediatric registry integrated with molecular-genetic data, which is essential for the transition toward precision oncology and the strategic allocation of resources to mitigate the long-term socio-economic impact of these malignancies on the population.

## 1 Introduction

Pediatric malignancies constitute a rare but clinically distinct group of over 100 different types, characterized by heterogeneous diagnostic profiles, complex molecular backgrounds, and divergent clinical trajectories (1). According to recent data from the Global Cancer Observatory, approximately 24,089 new cancer cases are diagnosed annually among the children and adolescent population (0–19 years) in Europe (2). According to the general annual statistical bulletin of the Polish National Cancer Registry (PNCR) 1,229 new cases were recorded in this age group in Poland in 2023, accounting for 0.63% of the total national cancer incidence, with 782 cases occurring in the 0–14 age cohort (3).

Despite the significant global burden of childhood cancer, there is a profound international disparity in the availability and quality of epidemiological surveillance. On a global scale, there is a critical shortage of high-quality, granular epidemiological data specifically focused on pediatric populations (4). Most existing surveillance systems are extensions of adult registries, which frequently fail to capture the unique classifications and late-effect profiles intrinsic to pediatric oncology. Furthermore, the absence of dedicated, internationally standardized pediatric cancer registries hinders the comparative analysis of incidence trends and the evaluation of therapeutic outcomes across different socio-economic regions (4, 5).

The biological paradigm of pediatric oncology differs fundamentally from adult carcinogenesis. While adult malignancies are largely driven by cumulative environmental exposures and lifestyle-related somatic mutations, pediatric neoplasms primarily result from developmental dysregulation and germline or early somatic genetic alterations (6). This distinct etiology necessitates specialized epidemiological surveillance frameworks that incorporate age-specific histopathological and molecular genetic classifications, which differ substantially from those applied to adult populations.

In the Polish healthcare landscape, the Polish National Cancer Registry (PNCR) serves as the primary instrument for monitoring the incidence and mortality of malignant neoplasms through a unified nationwide database. A significant development in 2023 was the implementation of the Polish Onco-Hematological Registry (PROH) within the PNCR framework. As a specialized quality registry, PROH collects comprehensive longitudinal data on hematological malignancies, whereas data on solid tumors in children continue to be recorded within the standard epidemiological scope of the PNCR (3).

The establishment of such systems is a critical step toward mitigating the data gaps identified in global oncology. A dedicated pediatric registry framework allows for the integration of ultra-rare condition surveillance, long-term survivorship monitoring, and the identification of novel prognostic biomarkers. Such granular data are indispensable for the strategic allocation of healthcare resources and the refinement of evidence-based oncological policies.

In alignment with the National Oncology Strategy, this study leverages the comprehensive resources of the PNCR to analyze the frequency and incidence rates of newly diagnosed pediatric malignancies in Poland for the year 2023. By evaluating morbidity trends across specific age and gender cohorts, we aim to address the existing gaps in pediatric epidemiological data and underscore the pivotal role of the PNCR as the cornerstone for clinical and translational research in Poland.

## 2 Material and methods

### 2.1 Data source

The primary data were derived from the PNCR. As a standardized, population-based registry, the PNCR provides a robust framework for monitoring cancer incidence across Poland. Reporting of cancer cases is mandatory for all healthcare providers (including both public and private sectors). The operating standards and data collection protocols of the PNCR have been documented extensively in previous literature (3). To ensure data integrity and prevent duplicate entries, the registry utilizes the Polish personal identification number (PESEL, Powszechny Elektroniczny System Ewidencji Ludności), which excludes the risk of double coding or duplicate cancer cases. The structure of the Polish population by sex was obtained from the Central Statistical Office on 30 June 2023, according to permanent domicile.

### 2.2 Population and age definition

In accordance with international standards, including those established by the study Benchmarking of Childhood Cancer Survival by Stage (Benchista) and the Surveillance, Epidemiology, and End Results (SEER) Program, the pediatric population was defined as individuals aged 0–19 years to enable cross-national comparisons (7, 8). To provide a more granular epidemiological perspective, a separate analysis was conducted for the 0–14 age group, which corresponds to the classical definition of childhood cancer.

### 2.3 Case selection and classification

The study included all primary malignancies diagnosed in 2023. Cases were coded according to the International Classification of Diseases for Oncology, Third Edition (ICD-O-3.2) (9). In accordance with standard cancer registration guidelines, transformations observed in hematological malignancies (e.g., leukemic transformation of lymphoma) were recorded under the primary diagnosis to avoid artificial inflation of incidence rates. For comparative consistency, all cases were categorized according to the International Classification of Childhood Cancer (ICCC-3) system, which is based on morphological type and anatomical site rather than primary site alone (10). Because reporting to the PNCR is mandatory and utilizes the unique national identification number (PESEL), core demographic variables such as age and sex are complete. Cases with entirely missing primary site or morphology codes that could not be mapped precisely to specific ICCC-3 categories were classified as “Other and unspecified malignant neoplasms” (Group XII) rather than excluded, ensuring the total cancer burden was not artificially underestimated.

### 2.4 Statistical analysis

For better readability of rare pediatric events, all incidence rates were calculated per 1,000,000 person-years. The cross-sectional analysis for 2023 was based on crude incidence rates. Conversely, for the longitudinal time-trend analysis (2000–2023), age-standardized rates were calculated based on the Revised European Standard Population (ESP2013) (11) to ensure temporal comparability. Because childhood cancers are rare and subject to random annual fluctuations, a 3-year moving average was applied to the trend data to smooth out short-term statistical noise and reveal clearer long-term trajectories.

Time-trend analysis of incidence was conducted for the period 2000–2023 utilized a 3-year moving average to filter out noise and mitigate the impact of outliers. Gender-specific disparities were evaluated using the Incidence Rate Ratio (IRR), which was calculated as the ratio of the incidence rate in boys to the incidence rate in girls, assuming a Poisson distribution for the occurrence of cancer cases. The 95% confidence intervals (95% CI) and exact *P*-values were derived via Wald's method on log-transformed data (12). Statistical significance was set at *P* < 0.05. All statistical analyses were performed using R software version 4.4.2 (R Core Team/R Foundation for Statistical Computing, Vienna, Austria) (Pile of Leaves).

### 2.5 Ethical considerations

In accordance with Polish legislation regarding public health surveillance, the use of aggregated, de-identified PNCR data for statistical and scientific purposes does not require individual informed consent. The study was conducted in strict compliance with data protection regulations to ensure the complete confidentiality of all subjects.

## 3 Result

### 3.1 General incidence overview

In 2023, the PNCR recorded 1,229 new cases of malignant neoplasms (C00-C96) in the entire 0–19 age group (crude incidence rate 161.4 per million). When analyzed by distinct age cohorts, 782 new cases were identified in the childhood group (0–14 years; crude rate 136.1 per million), and 447 cases were recorded in the adolescent group (15–19 years; crude rate 239.3 per million; Table 1). The total cancer burden showed no significant gender disparity in either the childhood 0–14 group (IRR 1.10; 95% CI: 0.96–1.27; *P* = 0.181) or the adolescent 15–19 group (IRR 0.87; *P* = 0.129). Consequently, the aggregate 0–19 cohort was similarly balanced (IRR 1.01; 95% CI: 0.90–1.13; *P* = 0.864; Table 2).

**Table 1:** Numbers and crude rates (per 1 million population) of malignant neoplasms (C00-C96) among children, Poland 2023.

Age	Boys		Girls		Overall	
	No	Crude rate	No	Crude rate	No	Crude rate
0–4	161	185.0	136	164.7	297	175.1
5–9	108	106.3	98	101.9	206	104.2
10–14	151	142.0	128	127.0	279	134.7
15–19	213	222.5	234	256.9	447	239.3
0–14	420	142.4	362	129.5	782	136.1
0–19	633	162.0	596	160.8	1,229	161.4

No, number.

**Table 2:** Incidence of malignant neoplasms (C00-C96) among children aged 0–19 according to the International Classification of Childhood Cancer (ICCC) classification, Poland 2023.

ICCC group	Overall		Boys		Girls		IRR (boys to girls)	95% CI	*P*-value
	No	Crude rate	No	Crude rate	No	Crude rate			
Overall	1,229	161.4	633	162.0	596	160.8	1.01	0.90–1.13	0.864
I. Leukemias. Myeloproliferative and myelodysplastic diseases	338	44.4	185	47.4	153	41.3	1.15	0.93–1.42	0.196
(a) Lymphoid leukemias	265	34.8	143	36.6	122	32.9	1.11	0.87–1.41	0.397
(b) Acute myeloid leukemias	52	6.8	28	7.2	24	6.5	1.11	0.64–1.92	0.710
(c) Chronic myeloproliferative diseases	9	1.2	5	1.3	4	1.1	1.19	0.32–4.43	0.795
(d) Myelodysplastic syndrome and other myeloproliferative diseases	2	0.3	2	0.5	–	–	–	–	–
(e) Unspecified and other specified leukemias	10	1.3	7	1.8	3	0.8	2.21	0.57–8.55	0.250
II. Lymphomas and reticuloendothelial neoplasms	205	26.9	117	30.0	88	23.7	1.26	0.96–1.66	0.097
(a) Hodgkin lymphomas	111	14.6	57	14.6	54	14.6	1.00	0.69–1.45	1.000
(b) Non-Hodgkin lymphomas (except Burkitt lymphoma)	46	6.0	25	6.4	21	5.7	1.13	0.63–2.02	0.681
(c) Burkitt lymphoma	31	4.1	26	6.7	5	1.3	4.93	1.89–12.84	0.001^*^
(d) Miscellaneous lymphoreticular neoplasms	17	2.2	9	2.3	8	2.2	1.07	0.41–2.77	0.889
(e) Unspecified lymphomas	–	–	–	–	–	–	–	–	–
III. CNS and miscellaneous intracranial and intraspinal neoplasms	136	17.9	74	18.9	62	16.7	1.13	0.81–1.58	0.472
(a) Ependymomas and choroid plexus tumor	15	2.0	9	2.3	6	1.6	1.42	0.51–3.99	0.504
(b) Astrocytomas	30	3.9	15	3.8	15	4.0	0.95	0.46–1.94	0.888
(c) Intracranial and intraspinal embryonal tumors	28	3.7	17	4.4	11	3.0	1.47	0.69–3.14	0.319
(d) Other gliomas	38	5.0	19	4.9	19	5.1	0.95	0.50–1.79	0.874
(e) Other specified intracranial and intraspinal neoplasms	5	0.7	–	–	5	1.3	–	–	–
(f) Unspecified intracranial and intraspinal neoplasms	20	2.6	14	3.6	6	1.6	2.21	0.85–5.75	0.104
IV. Neuroblastoma and other peripheral nervous cell tumors	49	6.4	21	5.4	28	7.6	0.71	0.40–1.25	0.238
(a) Neuroblastoma and ganglioneuroblastoma	44	5.8	21	5.4	23	6.2	0.87	0.48–1.40	0.610
(b) Other peripheral nervous cell tumors	5	0.7	–	–	5	1.3	–	–	–
V. Retinoblastoma	17	2.2	6	1.5	11	3.0	0.52	0.19–1.41	0.200
VI. Renal tumors	54	7.1	25	6.4	29	7.8	0.82	0.48–1.40	0.467
(a) Nephroblastoma and other non-epithelial renal tumors	40	5.3	21	5.4	19	5.1	1.05	0.57–1.95	0.876
(b) Renal carcinomas	6	0.8	1	0.3	5	1.3	0.19	0.02–1.63	0.138
(c) Unspecified malignant renal tumors	8	1.1	3	0.8	5	1.3	0.57	0.14–2.39	0.437
VII. Hepatic tumors	7	0.9	3	0.8	4	1.1	0.71	0.16–3.17	0.653
(a) Hepatoblastoma and mesenchymal tumors of liver	3	0.4	2	0.5	1	0.3	1.90	0.17–20.96	0.601
(b) Hepatic carcinomas	2	0.3	1	0.3	1	0.3	0.95	0.06–15.19	0.971
(c) Unspecified malignant hepatic tumors	2	0.3	–	–	2	0.5	–	–	–
VIII. Malignant bone tumors	78	10.2	47	12.0	31	8.4	1.44	0.92–2.27	0.113
(a) Osteosarcomas	39	5.1	22	5.6	17	4.6	1.23	0.65–2.32	0.523
(b) Chondrosarcomas	7	0.9	6	1.5	1	0.3	5.69	0.69–47.26	0.106
(c) Ewing tumor and related sarcomas of bone	24	3.2	12	3.1	12	3.2	0.95	0.43–2.12	0.900
(d) Other specified malignant bone tumors	3	0.4	3	0.8	–	–	–	–	–
(e) Unspecified malignant bone tumors	5	0.7	4	1.0	1	0.3	3.80	0.43–34.00	0.230
IX. Soft tissue and other extraosseous sarcomas	77	10.1	45	11.5	32	8.6	1.33	0.85–2.09	0.213
(a) Rhabdomyosarcomas	28	3.7	16	4.1	12	3.2	1.27	0.60–2.69	0.531
(b) Fibrosarcomas. peripheral nerve sheath tumors and other fibrous neoplasms	10	1.3	7	1.8	3	0.8	2.21	0.57–8.55	0.250
(c) Kaposi sarcoma	–	–	–	–	–	–	–	–	–
(d) Other specified soft tissue sarcomas	27	3.5	17	4.4	10	2.7	1.61	0.74–3.52	0.231
(e) Unspecified soft tissue sarcomas	12	1.6	5	1.3	7	1.9	0.68	0.22–2.14	0.506
X. Germ cell tumors. Trophoblastic tumors and neoplasms of gonads	65	8.5	40	10.2	25	6.7	1.52	0.92–2.51	0.102
(a) Intracranial and intraspinal germ cell tumors	9	1.2	6	1.5	3	0.8	1.90	0.48–7.60	0.362
(b) Malignant extracranial and extragonadal germ cell tumors	7	0.9	4	1.0	3	0.8	1.27	0.28–5.68	0.755
(c) Malignant gonadal germ cell tumors	36	4.7	29	7.4	7	1.9	3.93	1.72–8.97	0.001^*^
(d) Gonadal carcinomas	6	0.8	–	–	6	1.6	–	–	–
(e) Other and unspecified malignant gonadal tumors	7	0.9	1	0.3	6	1.6	0.16	0.02–1.33	0.086
XI. Other malignant epithelial neoplasms and malignant melanomas	168	22.1	54	13.8	114	30.8	0.45	0.33–0.62	<0.0001^*^
(a) Adrenocortical carcinomas	5	0.7	1	0.3	4	1.1	0.24	0.03–2.15	0.190
(b) Thyroid carcinomas	98	12.9	17	4.4	81	21.9	0.20	0.12–0.34	<0.0001^*^
(c) Nasopharyngeal carcinomas	2	0.3	2	0.5	–	–	–	–	–
(d) Malignant melanomas	16	2.1	11	2.8	5	1.3	2.09	0.73–6.02	0.170
(e) Skin carcinomas	6	0.8	2	0.5	4	1.1	0.47	0.09–2.57	0.377
(f) Other and unspecified carcinomas	41	5.4	21	5.4	20	5.4	1.00	0.54–1.85	1.000
XII. Other and unspecified malignant neoplasms	35	4.6	16	4.1	19	5.1	0.80	0.41–1.56	0.513
(a) Other specified malignant tumors	8	1.1	4	1.0	4	1.1	0.95	0.24–3.80	0.942
(b) Other unspecified malignant tumors	27	3.5	12	3.1	15	4.0	0.76	0.36–1.62	0.474

^*^Statistically significant (*P* < 0.05).

No, number; IRR, Incidence Rate Ratio; CI, confident interval; CNS, central nervous system.

### 3.2 Age-specific incidence patterns

**Figure 1:**
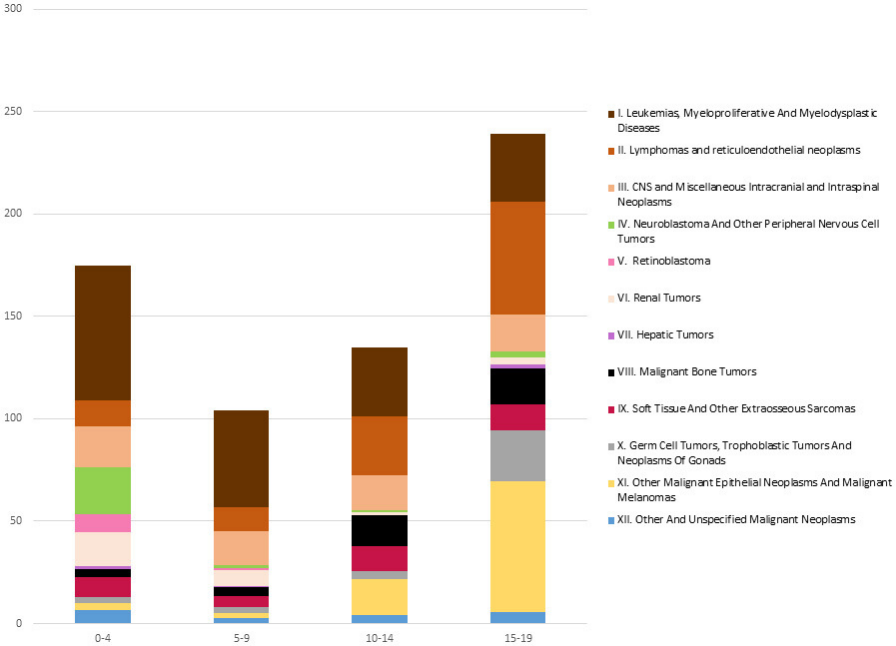
Age-specific crude incidence rates of malignant neoplasms by International Classification of Childhood Cancer (ICCC-3) group and age cohort (Poland, 2023), CNS, central nervous system.

Incidence followed a bimodal distribution, peaking in children aged 0–4 years (175.1 per million) and adolescents aged 15–19 years (239.3 per million). The lowest rate was observed in the 5–9 age group (104.2 per million), while the 10–14 cohort reached 134.7 per million (Table 1, Figure 1).

### 3.3 Gender-specific Incidence and Rate Ratios (IRR)

Regarding the distribution of specific malignancies, leukaemias (Group I) constituted the primary diagnostic entity with a crude incidence rate of 44.4 per million in the 0–19 cohort and 48.0 per million in the 0–14 group, exhibiting no statistically significant gender disparity (*P* ≥ 0.05).

In contrast, lymphomas and reticuloendothelial neoplasms (Group II) demonstrated a significant male predominance within the 0–14 population (IRR 1.67; *P* = 0.013; Table 3). This trend was largely driven by Burkitt lymphoma (IIc), which showed extreme male predilection in the childhood cohort (0–14 years; IRR 10.43, *P* = 0.001; Table 3) as well as a non-significant male excess in the adolescent group (15–19 years; IRR 1.27, *P* = 0.754). The aggregate IRR for the combined 0–19 population was 4.93 (*P* = 0.001; Table 2).

**Table 3:** Incidence of malignant neoplasms (C00-C96) among children aged 0–14 according to the International Classification of Childhood Cancer (ICCC) classification, Poland 2023.

ICCC group	Overall		Boys		Girls		IRR (boys to girls)	95% CI	*P*-value
	No	Crude rate	No	Crude rate	No	Crude rate			
Overall	782	136.1	420	142.4	362	129.5	1.10	0.96–1.27	0.181
I. Leukemias. Myeloproliferative and myelodysplastic diseases	276	48.0	150	50.9	126	45.1	1.13	0.89–1.43	0.312
(a) Lymphoid leukemias	225	39.2	120	40.7	105	37.6	1.08	0.83–1.41	0.569
(b) Acute myeloid leukemias	37	6.4	20	6.8	17	6.1	1.12	0.58–2.13	0.733
(c) Chronic myeloproliferative diseases	3	0.5	2	0.7	1	0.4	1.90	0.16–19.30	0.599
(d) Myelodysplastic syndrome and other myeloproliferative diseases	2	0.3	2	0.7	0	0.0	–	–	–
(e) Unspecified and other specified leukemias	9	1.6	6	2.0	3	1.1	1.90	0.45–7.27	0.365
II. Lymphomas and reticuloendothelial neoplasms	102	17.8	65	22.0	37	13.2	1.67	1.11–2.50	0.0131^*^
(a) Hodgkin lymphomas	38	6.6	22	7.5	16	5.7	1.30	0.69–2.51	0.426
(b) Non-Hodgkin lymphomas (except Burkitt lymphoma)	24	4.2	13	4.4	11	3.9	1.12	0.51–2.52	0.781
(c) Burkitt lymphoma	24	4.2	22	7.5	2	0.7	10.43	2.52–45.56	0.0013^*^
(d) Miscellaneous lymphoreticular neoplasms	16	2.8	8	2.7	8	2.9	0.95	0.35–2.48	0.918
(e) Unspecified lymphomas	0	0.0	0	0.0	0	0.0	–	–	–
III. CNS and miscellaneous intracranial and intraspinal neoplasms	102	17.8	56	19.0	46	16.5	1.15	0.78–1.70	0.482
(a) Ependymomas and choroid plexus tumor	10	1.7	6	2.0	4	1.4	1.42	0.40–5.06	0.589
(b) Astrocytomas	21	3.7	12	4.1	9	3.2	1.26	0.54–3.04	0.599
(c) Intracranial and intraspinal embryonal tumors	25	4.4	15	5.1	10	3.6	1.42	0.64–3.15	0.388
(d) Other gliomas	25	4.4	12	4.1	13	4.6	0.88	0.41–1.95	0.748
(e) Other specified intracranial and intraspinal neoplasms	4	0.7	0	0.0	4	1.4	–	–	–
(f) Unspecified intracranial and intraspinal neoplasms	17	3.0	11	3.7	6	2.1	1.74	0.65–4.76	0.275
IV. Neuroblastoma and other peripheral nervous cell tumors	44	7.7	21	7.1	23	8.2	0.87	0.48–1.56	0.643
(a) Neuroblastoma and ganglioneuroblastoma	44	7.7	21	7.1	23	8.2	0.87	0.48–1.56	0.643
(b) Other peripheral nervous cell tumors	0	0.0	0	0.0	0	0.0	–	–	–
V. Retinoblastoma	17	3.0	6	2.0	11	3.9	0.52	0.19–1.39	0.198
VI. Renal tumors	47	8.2	22	7.5	25	8.9	0.83	0.48–1.49	0.518
(a) Nephroblastoma and other non-epithelial renal tumors	36	6.3	18	6.1	18	6.4	0.95	0.50–1.83	0.877
(b) Renal carcinomas	3	0.5	1	0.3	2	0.7	0.47	0.04–4.73	0.535
(c) Unspecified malignant renal tumors	8	1.4	3	1.0	5	1.8	0.57	0.13–2.32	0.444
VII. Hepatic tumors	4	0.7	2	0.7	2	0.7	0.95	0.14–7.10	0.959
(a) Hepatoblastoma and mesenchymal tumors of liver	3	0.5	2	0.7	1	0.4	1.90	0.16–19.30	0.599
(b) Hepatic carcinomas	0	0.0	0	0.0	0	0.0	–	–	–
(c) Unspecified malignant hepatic tumors	1	0.2	0	0.0	1	0.4	–	–	–
VIII. Malignant bone tumors	45	7.8	26	8.8	19	6.8	1.30	0.72–2.34	0.382
(a) Osteosarcomas	23	4.0	13	4.4	10	3.6	1.23	0.54–2.79	0.620
(b) Chondrosarcomas	2	0.3	2	0.7	0	0.0	–	–	–
(c) Ewing tumor and related sarcomas of bone	16	2.8	8	2.7	8	2.9	0.95	0.35–2.48	0.918
(d) Other specified malignant bone tumors	0	0.0	0	0.0	0	0.0	–	–	–
(e) Unspecified malignant bone tumors	4	0.7	3	1.0	1	0.4	2.84	0.26–24.03	0.366
IX. Soft tissue and other extraosseous sarcomas	53	9.2	32	10.9	21	7.5	1.44	0.84–2.52	0.193
(a) Rhabdomyosarcomas	23	4.0	13	4.4	10	3.6	1.23	0.54–2.79	0.620
(b) Fibrosarcomas. peripheral nerve sheath tumors and other fibrous neoplasms	5	0.9	3	1.0	2	0.7	1.42	0.24–8.55	0.700
(c) Kaposi sarcoma	0	0.0	0	0.0	0	0.0	–	–	–
(d) Other specified soft tissue sarcomas	18	3.1	12	4.1	6	2.1	1.90	0.73–5.20	0.199
(e) Unspecified soft tissue sarcomas	7	1.2	4	1.4	3	1.1	1.26	0.28–5.69	0.763
X. Germ cell tumors. Trophoblastic tumors and neoplasms of gonads	19	3.3	9	3.1	10	3.6	0.85	0.35–2.12	0.723
(a) Intracranial and intraspinal germ cell tumors	7	1.2	4	1.4	3	1.1	1.26	0.28–5.69	0.763
(b) Malignant extracranial and extragonadal germ cell tumors	5	0.9	3	1.0	2	0.7	1.42	0.24–8.55	0.700
(c) Malignant gonadal germ cell tumors	3	0.5	2	0.7	1	0.4	1.90	0.16–19.30	0.599
(d) Gonadal carcinomas	2	0.3	0	0.0	2	0.7	–	–	–
(e) Other and unspecified malignant gonadal tumors	2	0.3	0	0.0	2	0.7	–	–	–
XI. Other malignant epithelial neoplasms and malignant melanomas	48	8.4	18	6.1	30	10.7	0.57	0.32–1.02	0.057
(a) Adrenocortical carcinomas	4	0.7	1	0.3	3	1.1	0.32	0.03–2.62	0.317
(b) Thyroid carcinomas	22	3.8	4	1.4	18	6.4	0.21	0.07–0.65	0.006^*^
(c) Nasopharyngeal carcinomas	0	0.0	0	0.0	0	0.0	–	–	–
(d) Malignant melanomas	6	1.0	4	1.4	2	0.7	1.90	0.37–10.92	0.457
(e) Skin carcinomas	2	0.3	0	0.0	2	0.7	–	–	–
(f) Other and unspecified carcinomas	14	2.4	9	3.1	5	1.8	1.71	0.58–5.14	0.334
XII. Other and unspecified malignant neoplasms	25	4.4	13	4.4	12	4.3	1.03	0.47–2.24	0.941
(a) Other specified malignant tumors	6	1.0	3	1.0	3	1.1	0.95	0.18–4.50	0.949
(b) Other unspecified malignant tumors	19	3.3	10	3.4	9	3.2	1.05	0.43–2.61	0.916

^*^Statistically significant (*P* < 0.05).

No, number; IRR, Incidence Rate Ratio; CI, confident interval; CNS, central nervous system.

Central nervous system neoplasms (Group III) and other major solid tumors—including neuroblastoma (Group IV), renal tumors (Group VI), and malignant bone tumors (Group VIII)—showed no significant gender-based variation, with observed differences remaining within the limits of random fluctuation (*P* ≥ 0.05).

Conversely, marked disparities were identified in other categories: malignant gonadal germ cell tumors (Xc) exhibited a significant male predilection (IRR 3.93; *P* = 0.001; Table 2). The most pronounced female predominance was observed in Group XI (other malignant epithelial neoplasms and melanomas). This female bias was primarily driven by thyroid carcinomas (XIb), which demonstrated highly significant disparities in both the childhood group (0–14 years; IRR 0.21, *P* = 0.006; Table 3) and the adolescent group (15–19 years; IRR 0.20, *P* < 0.0001). The overall IRR for thyroid carcinomas in the combined 0–19 cohort was 0.20 (*P* < 0.0001; Table 2).

Detailed comparative data for both the 0–19 and 0–14 age groups are presented in the Tables 2, 3.

### 3.4 Long-term incidence trends

**Figure 2:**
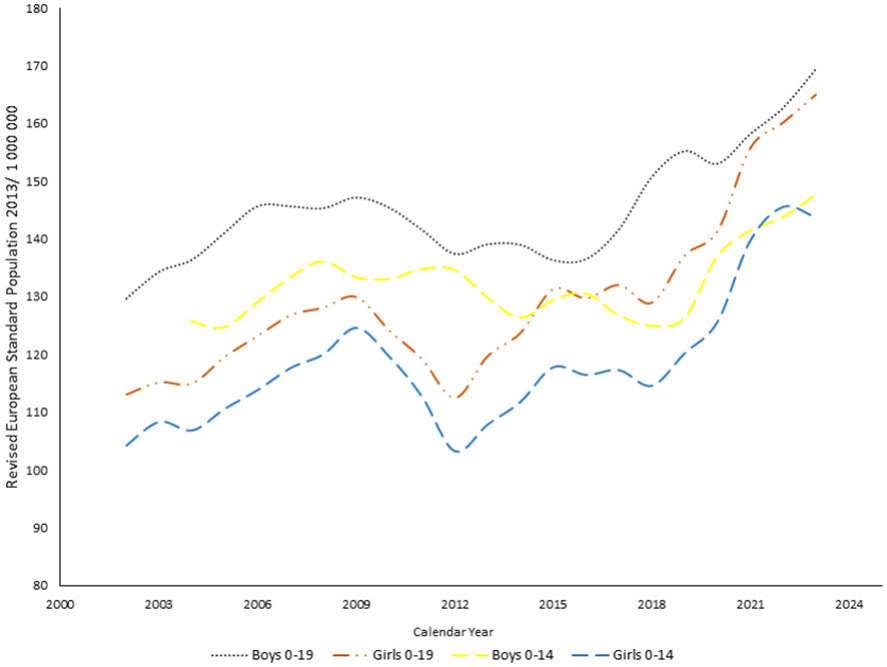
Long-term trends in age-standardized incidence rates of malignant neoplasms (C00-C96) among children and adolescents aged 0–19 (Poland, 2000–2023); ESP2013 – Revised European Standard Population 2013.

Descriptive analysis suggests an upward trajectory in incidence rates for all genders over time. It is important to note that this trend analysis is purely descriptive and does not employ formal regression models. The age-standardized rate (ESP2013) rose from around 110–120 per 1,000,000 in 2000 to over 160 per 1,000,000 in 2023. Throughout the analyzed period, the incidence rate was consistently higher in boys than in girls, although the trends converged noticeably between 2019 and 2023. An observed localized peak in rates occurred in the 2021–2022 period (Figure 2). This pattern coincides chronologically with the post-pandemic period and may suggest a compensatory “catching-up” effect related to diagnostic delays when access to healthcare was limited, though formal statistical testing is required to confirm this hypothesis.

A comparable upward trajectory was identified within the 0–14 age group. In this cohort, the incidence rate for boys rose from 131.0 to 163.1 per 1,000,000 between 2000 and 2021, while the rate for girls increased from 95.7 to 155.1 per 1,000,000. Notably, the gender gap, which was highly pronounced in the early 2000s, narrowed considerably toward the end of the study period. For instance, in 2022, rates reached 147.3 and 146.1 per 1,000,000 for males and females, respectively, indicating a near-unification of gender-specific incidence. Despite the inherent stochastic variability associated with small sub-population sizes, the consistent upward trend underscores a growing epidemiological challenge in pediatric oncology that transcends gender boundaries.

## 4 Discussion

The 2023 pediatric cancer incidence in Poland (161.4 per million for 0–19; 136.1 per million for 0–14) aligns with the upper European average of 155–165 per million, reflecting high diagnostic efficiency and robust registry infrastructure (4, 13). The descriptive upward trajectory since 2000, with an observed peak in 2021–2022, coincides chronologically with COVID-19 pandemic disruptions. While systematic reviews confirm significant delays and reductions in diagnoses during the initial pandemic waves (14), our observational data may suggest that these delayed cases were subsequently captured by the registry. This potential “catching-up” effect is visually evident in the 0–14 age group, where the incidence among boys reached a peak of 163.1 per million in 2021; however, establishing a definitive causal link to the pandemic would require formal interrupted time-series analysis.

Leukaemias remain the predominant pediatric malignancy, with a crude incidence rate of 48.0 per million in the 0–14 group (Table 3) and 33.2 per million in the 15–19 adolescent group. The aggregate rate for the entire 0–19 cohort stood at 44.4 per million (Table 2). This stable dominance aligns with global epidemiological profiles and underscores the central role of specialized hemato-oncological care in pediatric health systems (4).

While the aggregate cancer burden was balanced between genders (IRR 1.01), significant biological divergences emerged in specific subtypes. The fivefold higher risk of thyroid carcinoma in females (0–19 IRR: 0.2; 0–14 IRR: 0.21) suggests a critical role for hormonal factors during puberty, though potential diagnostic over-surveillance via ultrasonography must be considered (15).

Conversely, a marked male predominance was observed in lymphomas, reaching statistical significance in the 0–14 age group (IRR 1.67, *P* = 0.013). This disparity was most pronounced in Burkitt lymphoma, with an extreme male predilection in the younger cohort (IRR 10.43). These findings, along with the male predominance in gonadal germ cell tumors (IRR 3.93), align with established biological peaks and underscore the necessity for sex-stratified screening protocols (16, 17).

The consistency of the PNCR data with European indicators underscores its role as a high-quality resource for epidemiological surveillance. Methodological variations in case reporting and classification often account for disparities observed between national registries. A significant strength of the current analysis is the integration of the PROH, which records all oncological transformations within the primary diagnosis. This approach prevents the artificial overestimation of incidence rates by eliminating potential “duplicates” in hemato-oncological cases, which constitute approximately 44% of the pediatric cancer burden in this study.

Furthermore, our findings must be considered in the context of evolving classification systems. Periodic updates to the International Classification of Diseases for Oncology (ICD-O) can significantly impact incidence calculations; for instance, the reclassification of certain tumors from malignant (code /3) in ICD-O-3 to borderline (code /1) in subsequent versions (ICD-O-3.1/3.2) can lead to apparent shifts in epidemiological trends. The PNCR's adherence to the latest ICD-O-3.2 standards ensures that the 2023 data remains robust and comparable at an international level. This methodological rigor confirms the registry's representativeness and its value in evaluating the effectiveness of the national healthcare system and guiding preventive strategies in pediatric oncology.

### 4.1 Limitations and strengths of the study

The primary strength of this study lies in its population-based design, utilizing nationwide data from the PNCR to ensure comprehensive coverage of pediatric malignancies. Mandatory reporting across all healthcare sectors minimizes selection bias and enhances data completeness. Furthermore, the application of standardized international classification systems (ICD-O-3.2, ICCC-3) and age-standardization (ESP2013) facilitates robust global comparability. The granular, sex-specific analysis over a 23-year period provides a high-resolution epidemiological perspective, revealing subgroup patterns often masked in aggregate datasets.

Despite the registry's high coverage, international comparisons may be influenced by heterogeneous registration practices and classification nuances across different national systems. Furthermore, the descriptive and exploratory nature of this epidemiological analysis necessitates careful interpretation of the statistical tests. We performed numerous hypothesis tests across various tumor subtypes without applying statistical corrections for multiple comparisons, which inherently increases the risk of Type I errors (false positives). Additionally, the intrinsic rarity of specific pediatric oncological subtypes results in very small cohort sizes. For instance, while the extreme male predominance observed in Burkitt lymphoma is noteworthy (IRR = 10.43), this estimate is based on a minimal number of events, yielding statistically unstable estimates with notably wide confidence intervals. Therefore, the exact magnitude of the IRR and associated *P*-values for such ultra-rare subgroups should be interpreted with significant caution.

## 5 Conclusions

The 2023 epidemiological data confirm that pediatric cancer incidence in Poland aligns with established high-income European patterns, reflecting the robust diagnostic and surveillance capabilities of the national registry. Although children and adolescents constitute a declining demographic proportion of the European population due to sustained low fertility rates (with Poland's total fertility rate at 1.20 in 2023, significantly below the EU average of 1.38 and the replacement level of 2.1), the public health significance of this cohort remains paramount (18). The intrinsic rarity of these malignancies is counterbalanced by their profound impact on population health metrics. Pediatric cancers are characterized by a high burden of disability-adjusted life years and necessitate resource-intensive, lifelong survivorship monitoring (19).

From a socio-economic and public health perspective, in an aging society facing a demographic crisis, the loss of a child or their permanent disability represents an irreparable systemic loss. Importantly, high-efficiency pediatric oncology is one of the most economically effective healthcare interventions, as evidenced by its substantial social return on investment; comprehensive scale-up of treatment could avert millions of premature deaths, resulting in massive global lifetime productivity gains. Furthermore, the impact of a pediatric malignancy extends far beyond the individual patient; a diagnosis triggers a profound crisis for the entire family unit, imposing a significant psychological and functional burden on caregivers. This multi-generational impact, coupled with the potential for survivors to contribute decades of productivity to the economy, underscores the strategic obligation to preserve the country's diminishing human capital through precision diagnostics and optimized therapeutic strategies (20).

Significant gender-specific risks (most notably regarding thyroid carcinoma and Burkitt lymphoma) necessitate the implementation of sex-stratified clinical awareness (1) and diagnostic protocols. Furthermore, the high incidence rates observed among adolescents highlight a critical epidemiological transition, underscoring the importance of developing specialized AYA oncology programmes tailored to this cohort's distinct biological and psychosocial needs (19).

The Polish National Cancer Registry (PNCR) continues to provide high-quality, consolidated data that serves as the evidentiary cornerstone for the National Oncology Strategy (21). However, to optimize precision oncology and national therapeutic outcomes, the establishment of a dedicated pediatric cancer registry integrated with molecular-genetic parameters is highly recommended (19). Such a framework would not only allow for granular monitoring of long-term outcomes but also enable sophisticated cost-effectiveness analyses of novel therapies. This is essential to ensure that the quality of oncological care and the preservation of long-term health potential remain a top priority for national public health policy.

From a European policy perspective, the present findings highlight the critical importance of robust, population-based pediatric cancer registries as a cornerstone of evidence-based oncology. In line with the Europe's Beating Cancer Plan, strengthening data integration, including molecular and longitudinal survivorship data, should be prioritized to enable precision oncology and reduce disparities across Member States (22). In the context of declining fertility rates and aging populations, pediatric cancers represent a disproportionate loss of future human capital, underscoring their strategic relevance beyond healthcare alone (19).

## Data Availability

The datasets presented in this study can be found in online repositories. The names of the repository/repositories and accession number(s) can be found in the article/supplementary material.
